# Age- and Sex-Specific Patterns of Arterial Stiffness Assessed by Cardio–Ankle Vascular Index in Apparently Healthy Chinese Adults: A Cross-Sectional Study

**DOI:** 10.3390/metabo16050300

**Published:** 2026-04-29

**Authors:** Kai-Wen Hu, Bo-Li Cheng, Pin-Shi Ni, Zhuang-Zhi Wang, Fang-Hui Li

**Affiliations:** 1 Department of Orthopedic Surgery, Affiliated Drum Tower Hospital, Medical School of Nanjing University, 321 Zhongshan Road, Nanjing 210008, China; 21450331@life.hkbu.edu.hk; 2 Department of Sport, Physical Education and Health, Hong Kong Baptist University, Kowloon Tong, Hong Kong SAR 999077, China; 3 School of Sport Sciences, Nanjing Normal University, Nanjing 210023, China

**Keywords:** arterial stiffness, cardio–ankle vascular index (CAVI), early vascular aging, sex differences, metabolic risk factors

## Abstract

**Objective:** This study examined age- and sex-specific correlates of arterial stiffness, assessed by the cardio–ankle vascular index (CAVI), in apparently healthy Chinese adults using an anthropometric–metabolic–inflammatory framework, and descriptively compared subgroup association patterns across these domains. **Methods:** In this cross-sectional study, 525 apparently healthy Chinese adults aged 20–78 years were included. Regression models with age-by-indicator interaction terms were used to test whether the age–CAVI association varied across anthropometric, metabolic, and inflammatory indicators. Sex-adjusted analyses were applied to the overall sample, sex-stratified analyses were used to characterize sex-specific patterns, and the Benjamini–Hochberg false discovery rate correction was applied for multiple interaction tests. **Results:** CAVI increased progressively with age, with a steeper age–CAVI association after 50 years (*p* < 0.05). Notably, females showed a transient midlife elevation. Association patterns appeared to differ by sex. In the sex-stratified interaction analyses, anthropometric signals were more prominent in men, particularly for height (*p* < 0.01), whereas metabolic-related interaction signals were more evident in women, with triglycerides providing the clearest corresponding signal and low-density lipoprotein cholesterol (LDL-C) showing a weaker accompanying pattern; the C-reactive protein (CRP)-related contrast was not retained after additional adjustment for blood pressure and smoking. **Conclusions:** CAVI increased with age, with a steeper rise after midlife and a transient midlife elevation in women. The association patterns across anthropometric, metabolic, and inflammatory indicators appeared to differ by sex, with signals from the anthropometric domain appearing more evident in men and metabolic-related signals appearing more evident in women. These findings suggest that age- and sex-specific interpretation of CAVI may be informative in preventive health check-up settings.

## 1. Introduction

Arterial stiffness is a core manifestation of vascular aging and an important predictor of cardiovascular events [1,2,3]. With advancing age, elastin degradation, collagen accumulation, and endothelial dysfunction reduce arterial compliance, increase vascular tone, and impair the buffering capacity of large arteries, thereby elevating systolic pressure and potentially contributing to microvascular damage [4,5,6,7]. The cardio–ankle vascular index (CAVI), a measure of arterial stiffness that is relatively independent of current blood pressure, has become an important tool for assessing vascular aging and subclinical vascular damage [8,9,10]. Because CAVI is derived from pulse transmission along the heart-to-ankle arterial pathway, it is generally interpreted as reflecting stiffness of large conduit arteries, including the aorta and major lower-extremity arteries, rather than resistance (small) arterial stiffness [6,9,11]. The concept of Early Vascular Aging (EVA) highlights that arterial stiffness can be higher than expected for metrical age, even in young and middle-aged adults, often in association with metabolic abnormalities, chronic low-grade inflammation, obesity, and unhealthy lifestyles [12,13,14,15,16,17]. Within this framework, the key question is whether arterial stiffness deviates upward from age-expected levels. Accordingly, CAVI can be used as a quantitative metric of arterial stiffness to characterize age-stratified trajectories across adulthood, providing a reference for interpreting age-referenced variability in vascular aging among apparently healthy adults [18].

Beyond age, multiple biological processes have been linked to arterial stiffening. Dyslipidemia, such as elevated triglycerides (TG) and low-density lipoprotein cholesterol (LDL-C) together with reduced high-density lipoprotein cholesterol (HDL-C), has been associated with structural arterial changes, potentially through oxidative stress, endothelial dysfunction, and smooth muscle remodeling [5,19,20]. Chronic low-grade inflammation, often indexed by C-reactive protein (CRP), has been linked to extracellular matrix remodeling and is also associated with stiffening [19,21,22,23,24,25]. Mechanistically, correlates of arterial stiffness can be conceptualized as reflecting (1) body size–related structural loading and arterial geometry [20,26], (2) metabolic dysregulation linked to endothelial function and vascular remodeling [19,27], and (3) inflammatory processes associated with arterial wall and extracellular matrix remodeling [5,19]. Together, these domains represent three major and partially distinct biological pathways implicated in arterial stiffening, providing a parsimonious and interpretable structure to compare association patterns with CAVI across processes in apparently healthy adults. Based on these mechanistic considerations and prior literature, the present study grouped potential correlates of CAVI into three biological domains: an anthropometric domain (height, weight, body mass index (BMI), waist circumference, hip circumference, and pulse rate, reflecting body size and basic hemodynamic features) [26,28]; a metabolic domain (lipid and glycemic profiles, including TG, LDL-C, HDL-C, total cholesterol (TC), triglyceride-rich lipoprotein cholesterol (TRL-C), glycated hemoglobin (HbA1c), and fasting glucose (GLU)) [19,27,29,30,31]; and an inflammatory domain (CRP, reflecting chronic low-grade inflammation) [19,24,25]. This three-domain framework enables comparison of the relative strength of associations across distinct biological processes with CAVI within a unified structure.

Although age is widely recognized as the strongest correlate of CAVI, most existing studies have focused on older adults or patients with established cardiovascular disease [8], and evidence from healthy adults across different life stages remains limited. Moreover, men and women differ in metabolic profiles, hormonal status, and inflammatory responses, yet it is unclear whether these sex-specific characteristics are associated with key correlates of CAVI or whether the strength of associations across domains varies by age over the adult lifespan [32,33]. Previous studies in Japanese and, to a lesser extent, Chinese populations have reported age- and sex-related differences in CAVI, but most data come from specific regions or disease-based cohorts, and age- and sex-specific patterns in CAVI and their primary correlates among broadly defined, apparently healthy Chinese adults are still underexplored [10,11,25,30]. Therefore, the primary aim of this cross-sectional study was to characterize age- and sex-specific patterns of CAVI in apparently healthy Chinese adults. The secondary aim was to examine whether the age–CAVI association differed across subgroups defined by anthropometric, metabolic, and inflammatory indicators in the overall sample and in sex-stratified analyses. Together, these analyses provide descriptive evidence to support a more context-specific interpretation of CAVI in preventive health assessment.

## 2. Materials and Methods

### 2.1. Participants

Between December 2022 and June 2023, the research team screened 1328 self-reported healthy adults aged 20 to 78 years who underwent routine health examinations at Changzhou Sports Hospital, China. The selection criteria for the older adult population were based on the Chinese Health Standards for the Elderly (2022 edition) issued by the National Health Commission.

Through a comprehensive assessment that included physical examination, routine blood and urine analyses, biochemical tests (fasting glucose, lipid profile, liver and kidney function, and uric acid), electrocardiography, chest radiography, pulmonary function testing, echocardiography, and carotid ultrasonography, 525 individuals met the eligibility criteria and were included as apparently healthy participants. All participants provided written informed consent.

Based on the age distribution of the population in 2023, participants were categorized into five age groups: <30 years, 30–39 years, 40–49 years, 50–59 years, and ≥60 years, defined as Groups 1 through 5, respectively.

In the present study, “apparently healthy” was defined operationally as the absence of known major chronic disease, no major abnormalities on routine health examination, and no current use of medications likely to affect arterial stiffness or the measured cardiometabolic/inflammatory biomarkers. This definition does not imply that every biomarker was identical or strictly within a single optimal range in all participants; rather, inter-individual variation within the screened sample remained and was used for within-sample subgroup analyses.

### 2.2. Inclusion and Exclusion Criteria

(1) Inclusion criteria were as follows: no history of cardiovascular disease, cerebrovascular disease, renal disease, pulmonary disease, liver disease, rheumatic disease, chronic infection, or tumors; no history of psychological or consciousness disorders; no clinically diagnosed diabetes or hypertension; no obvious abnormalities in the head, neck, chest, abdomen, limbs, or nervous system; no major abnormalities in routine blood and urine tests, liver function, kidney function, fasting blood glucose, or blood lipid profiles; and no obvious abnormalities on chest radiography, echocardiography, and cervical vascular ultrasonography.

(2) Exclusion criteria: Patients with severe primary diseases such as liver, kidney, cardiovascular, cerebrovascular, and hematopoietic system diseases; individuals with mental disorders; severe intellectual disabilities; those reporting current use of medications that may affect arterial stiffness or cardiometabolic, inflammatory biomarkers (e.g., antihypertensive, lipid-lowering, glucose-lowering agents, systemic anti-inflammatory drugs/corticosteroids, or sex hormone therapy) and those with missing physical examination information.

After applying these criteria, a total of 525 participants were included in the final analysis.

### 2.3. Ethical Considerations

Ethical approval for this study was obtained from the Biomedical Research Ethics Committee of Nanjing Normal University (Approval No. NNU202310007). All participant data were coded to ensure confidentiality, and no monetary or material compensation was provided to any participant.

### 2.4. Arterial Stiffness Instruments and Methods

Arterial stiffness was assessed using the cardio–ankle vascular index (CAVI) with a fully automated vascular screening device (VP-1000, Colin Co., Ltd., Komaki, Japan). Participants rested in a supine position for at least 10 min in a quiet, temperature-controlled room before measurement. Cuffs were placed on both upper arms and ankles, a phonocardiographic sensor was positioned at the fourth intercostal space on the left sternal border, and electrocardiographic electrodes were attached to the wrists according to the manufacturer’s protocol. The device automatically recorded the required pulse wave and pressure signals and calculated CAVI. CAVI was calculated as CAVI = a × {(2ρ/ΔP) × ln(Ps/Pd) × pulse wave velocity (PWV)^2^} + b, where Ps = systolic pressure (mmHg), Pd = diastolic pressure (mmHg), ΔP = Ps − Pd, ρ = blood density (g/cm^3^), and a and b are calibration constants provided by the manufacturer. The mean of the left and right CAVI values was used for subsequent analyses.

### 2.5. Biochemical Measurements

Fasting venous blood samples were collected in the morning after an overnight fast at the Physical Examination Center of Changzhou Sports Hospital, Affiliated Sports Hospital of Nanjing Normal University (Changzhou, China) by trained staff. Blood for the lipid panel and CRP was collected into serum separator tubes, allowed to clot at room temperature, and centrifuged to obtain serum according to the center’s routine clinical standard operating procedure (SOP). Whenever possible, assays were performed on the day of collection as part of routine testing. If immediate analysis was not feasible, separated serum was kept at 4 °C for short-term storage and, when longer-term retention was required (e.g., re-testing/archival), aliquoted and stored at −80 °C until analysis, according to the laboratory SOP. HbA1c was measured from EDTA-anticoagulated whole blood using an automated enzymatic HbA1c assay, whereas serum TC and TG were measured using routine enzymatic assays, HDL-C and LDL-C were determined using direct homogeneous assays, GLU was measured in serum or plasma using a hexokinase-based method, and CRP was measured using immunoturbidimetry. These biochemical assays were performed on a BS-2000M automated chemistry analyzer (Mindray, Shenzhen, China). TRL-C was derived from the lipid panel as TC−LDL-C−HDL-C. Internal quality control and instrument calibration were conducted according to routine laboratory practice, and external quality assessment was performed when applicable.

### 2.6. Observation Indicators

(1) Anthropometric Indicators: Anthropometric and related hemodynamic indicators included height, weight, BMI, waist circumference, hip measurement, and pulse rate. These indicators were used to characterize body size, body-shape-related dimensions, and basic physiological features within the analytic framework of the present study.

(2) Metabolic Indicators: Metabolic status was assessed using lipid and glucose profiles, including TC, TG, LDL-C, HDL-C, TRL-C, GLU, and HbA1c.

(3) Inflammatory Indicator: Chronic low-grade inflammation was assessed using CRP.

### 2.7. Statistical Analysis

SPSS Statistics, version 26.0 (IBM Corp., Armonk, NY, USA) was used for statistical analysis, and analyses were performed using the available data for each variable. Continuous variables are presented as the mean ± standard deviation (SD). The distributions of continuous variables used in age-group comparisons were assessed using the Shapiro–Wilk test. Differences in CAVI and other continuous variables across age groups were analyzed using one-way analysis of variance (ANOVA). When the overall ANOVA was significant, post hoc pairwise comparisons were performed using Tukey’s honestly significant difference (HSD) test. Linear regression models were used to examine the association between age and CAVI, and age-by-indicator interaction terms were used to assess whether the age–CAVI association varied across physiological indicators in sequential models (Model 1, unadjusted; Model 2, adjusted for sex; Model 3, further adjusted for systolic and diastolic blood pressure (SBP and DBP); and Model 4, further adjusted for smoking status). For subgroup interaction analyses, anthropometric indicators were categorized into low and high subgroups using sample-based cut-off values, whereas biochemical indicators were categorized using indicator-specific cut-off values. In sex-stratified subgroup analyses, sex-specific subgroup ranges were applied separately within men and women. These subgroup definitions were used for descriptive subgroup interaction analyses and visualization only and were not intended to represent clinical diagnostic thresholds. Detailed subgroup definitions are summarized in Supplementary Table S4. Sex differences in the age–CAVI association were also examined. All statistical tests were two-sided, and statistical significance was set at *p* < 0.05.

To address multiplicity arising from multiple subgroups, we controlled the false discovery rate (FDR) using the Benjamini–Hochberg (BH) procedure across an overall family defined as all age-by-indicator interaction tests reported in the Results. Tests in other tables primarily reflect descriptive age-trend comparisons or baseline characteristic differences and do not belong to the age-indicator interaction-testing framework; therefore, they were not pooled into the interaction-test family for Benjamini–Hochberg false discovery rate (BH-FDR) adjustment. Raw interaction *p*-values and BH-adjusted *p*-values are provided in Supplementary Table S1, and results were considered significant after FDR if BH-adjusted *p* < 0.05. We pre-specified age-height, age-LDL-C, age-TG, and age-CRP as primary interaction hypotheses; all other interaction findings were interpreted as exploratory.

## 3. Results

### 3.1. Demographic Characteristics of Participants

Among the 525 participants included in the analysis, ages ranged from 20 to 78 years. There were 265 males (50.4%) with a mean age of 43.4 ± 10.8 years and 260 females (49.6%) with a mean age of 42.1 ± 10.5 years. The mean height and weight of males were 173.4 ± 6.8 cm and 83.9 ± 15.5 kg, respectively, whereas females had a mean height of 161.0 ± 5.6 cm and a mean weight of 66.2 ± 13.3 kg.

### 3.2. Age-Related Trajectories of CAVI and Sex Differences

**Figure 1:**
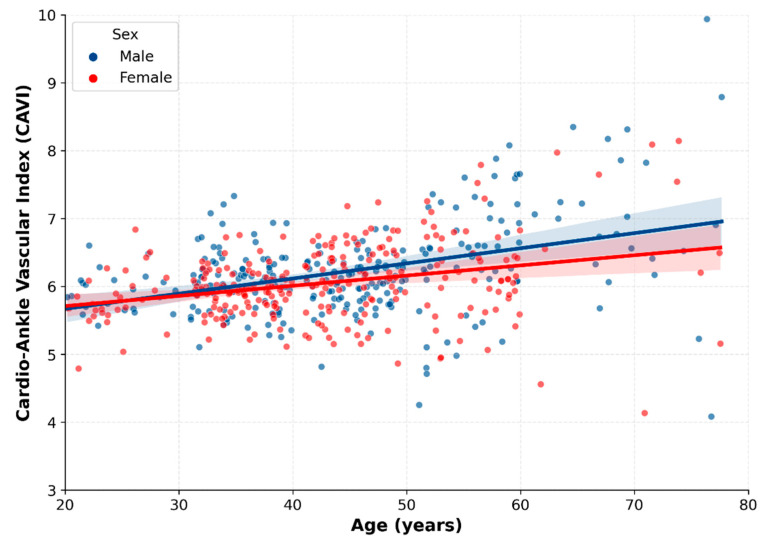
Age-related trajectories of arterial stiffness in healthy adults stratified by sex. Note: Scatter plot showing individual data points for males (blue circles) and females (red circles). Solid lines represent the linear regression trends for each sex, and shaded areas indicate the 95% confidence intervals. Cardio-ankle vascular index (CAVI) values demonstrate a progressive increase with age in both groups, with distinct trajectories observed between sexes.

Consistent with the age-related patterns of vascular aging, CAVI values showed a clear positive association with age across the entire cohort (Figure 1). As illustrated in Figure 1 and Table 1, this increasing trend was not strictly linear but appeared to become steeper after midlife: participants aged 50–59 years and those ≥60 years demonstrated significantly higher CAVI values compared to younger cohorts (*p* < 0.05), suggesting a potential change point in arterial stiffening. Regression analyses (Table 2) showed age to be the strongest correlate of arterial stiffness (β = 0.023, *p* < 0.0001).

Sex-stratified analyses revealed different age–CAVI association patterns. Males displayed a generally steeper overall age-related association (β = 0.026, 95% CI: 0.019–0.033) compared to females (β = 0.019, 95% CI: 0.012–0.026) (Table 3). However, visual inspection of the trajectories suggests a nonlinear pattern: while males maintained higher baseline stiffness values throughout early adulthood, female CAVI values showed a transient but notable midlife elevation, which appeared compatible with midlife physiological changes. Because menopausal status was not available in the current dataset, this midlife female pattern should be interpreted cautiously. Overall, the results indicate that the age–CAVI association appeared to differ by sex.

### 3.3. Associations of Anthropometric Factors with CAVI

**Figure 2:**
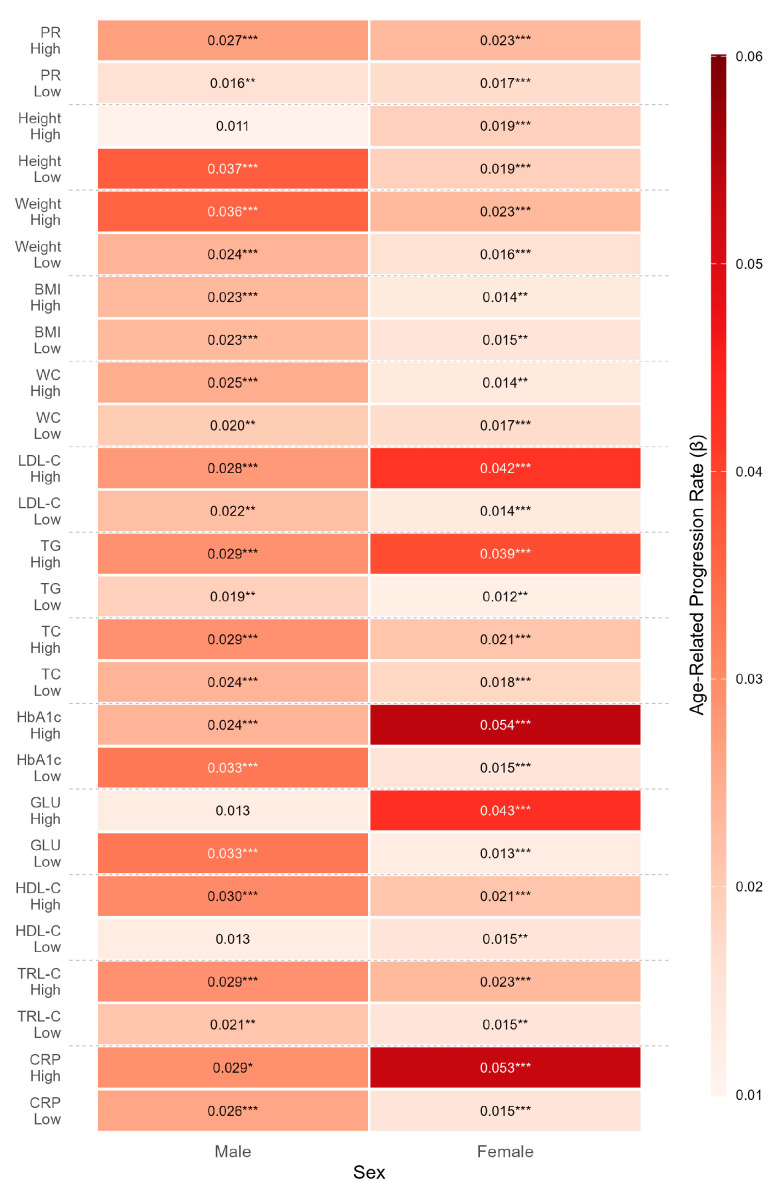
Sex-specific heatmap of subgroup-specific age–CAVI regression coefficients. Note: PR, pulse rate, BMI, body mass index, WC, Waist circumference, LDL-C, low-density lipoprotein cholesterol, TG, triglycerides, TC, total cholesterol, HbA1c, glycated hemoglobin, GLU, fasting glucose, HDL-C, high-density lipoprotein cholesterol, TRL-C, triglyceride-rich lipoprotein cholesterol, CRP, C-reactive protein. The heatmap displays the regression coefficients (β), representing the annual change in CAVI within each subgroup. Darker red indicates a larger age–CAVI coefficient. Asterisks denote the statistical significance of the regression coefficient within each subgroup (* *p* < 0.05, ** *p* < 0.01, *** *p* < 0.001) and do not indicate the significance of the interaction between high and low subgroups. In men, relatively larger coefficients were more often observed in anthropometric subgroups, particularly the low-height subgroup. In women, relatively larger coefficients were more often observed in several metabolic subgroups. BMI and waist circumference were included among the anthropometric indicators, but they did not emerge as major interaction signals in either sex.

To investigate whether anthropometric characteristics are associated with differences in the age–CAVI association, stratified regression analyses were performed with age as the independent variable and CAVI as the dependent variable. As shown in Table 4, height showed a significant interaction with age in relation to CAVI (*p* < 0.05 for interaction). Specifically, participants in the low-height group exhibited a steeper age–CAVI association in the sex-adjusted model (β = 0.029, 95% CI: 0.022–0.036) compared to those in the high-height group (β = 0.016, 95% CI: 0.008–0.024). This suggests that shorter stature is associated with a steeper age–CAVI association. In contrast, other anthropometric indicators, including weight, BMI, waist circumference, hip circumference, and pulse rate, did not show statistically significant interactions with age in relation to CAVI (*p* > 0.05). Additionally, a comparison of anthropometric characteristics across age groups (Table 5) showed expected age-related patterns, with height, weight, BMI, and pulse rate tending to be lower in older age groups, whereas the age-related pattern for waist circumference was less consistent. The sex-specific heatmap (Figure 2) further illustrates the relative prominence of the height-related pattern, particularly in men, in the descriptive subgroup analyses. The age-height interaction remained statistically significant after BH-FDR correction, whereas the remaining anthropometric interactions did not survive FDR correction (Supplementary Table S1).

### 3.4. Associations of Metabolic Factors with CAVI

Stratified analyses revealed that lipid profiles showed heterogeneous age interactions with the age-related trajectory of CAVI. After adjusting for sex (Model 2), TG, LDL-C, and HDL-C all showed significant interactions with age (Table 6); however, after additional adjustment for blood pressure and smoking, TG remained the clearest signal, whereas LDL-C was attenuated and HDL-C was no longer retained as a robust finding. Specifically, relatively higher lipid levels within the analytic sample were associated with a steeper age–CAVI association. As detailed in Table 6, the age-related regression coefficient was markedly higher in the high-TG group (β = 0.031) compared to the low-TG group (β = 0.014, *p* < 0.001). Similarly, the high-LDL-C group showed a steeper increase in CAVI in the sex-adjusted model (β = 0.030) than the low-LDL-C group (β = 0.016). These results suggest that, within this screened sample, relatively higher TG levels were consistently associated with a steeper age–CAVI association, whereas the corresponding pattern for LDL-C was weaker after additional adjustment. In contrast, although HDL-C showed a nominal age interaction in the sex-adjusted model, this interaction was further attenuated after additional adjustment and did not remain significant after BH-FDR correction and was therefore interpreted as exploratory (Supplementary Table S1). Regarding the age-related distribution of these metabolic markers (Table 7), LDL-C and TC showed a gradual increase with advancing age (*p* < 0.05), whereas age-related trends for TG and HDL-C were less consistent across the lifespan. The sex-specific patterns in these age interactions are further illustrated and summarized in Figure 2, as descriptive subgroup contrasts.

### 3.5. Associations of Inflammatory and Glycemic Factors with CAVI

Within the inflammatory domain, CRP showed an age-related interaction signal in Model 1 (with no adjustments). After adjusting for sex (Model 2), the interaction between CRP and age was highly significant (Table 8), but this pattern was not retained after additional adjustment for blood pressure and smoking. Notably, participants with elevated CRP levels showed the steepest age-related increase in CAVI in the sex-adjusted model (β = 0.042, 95% CI: 0.027–0.057), which was approximately twice the slope observed in the low-CRP group (β = 0.021, 95% CI: 0.016–0.026). This suggests that higher CRP was associated with a steeper age–CAVI association in this population in the earlier models. Importantly, the CRP–age interaction remained statistically significant after BH-FDR correction in the earlier model set, but was not retained in the updated models after additional adjustment (Supplementary Table S1).

In contrast, glycemic indicators, including HbA1c and GLU, showed weaker associations. As shown in Table 8, the interactions of HbA1c and GLU with age did not reach statistical significance (*p* > 0.05) in the overall cohort, which may relate to the study’s exclusion of individuals with clinically diagnosed diabetes. Regarding age-related trends (Table 9), both HbA1c and GLU levels showed a progressive increase across age groups (*p* < 0.05), consistent with age-related changes in glucose tolerance. Although the interactions appeared weak in the overall cohort, the sex-stratified analyses suggested that subgroup association patterns for these inflammatory and glycemic indicators differed between men and women, as illustrated in Figure 2.

### 3.6. Sex-Specific Association Patterns Across Domains

The sex-stratified coefficients underlying Figure 2 are presented numerically in Table 10. These results suggested that subgroup association patterns related to the age–CAVI relationship may differ between men and women.

As shown in the left column of Figure 2 and Table 10, relatively larger age–CAVI coefficients in men were more often observed in the anthropometric domain, particularly for height. The low-height subgroup showed the steepest age–CAVI association in men (β = 0.037), whereas the high-weight subgroup also showed a relatively large coefficient in the heatmap (β = 0.036), although the age-weight interaction was not statistically significant. However, in men, neither BMI (*p* = 0.9533) nor waist circumference (*p* = 0.4629) showed a statistically significant interaction with age in relation to CAVI. These findings suggest that anthropometric interaction signals appeared more prominent than several metabolic or inflammatory signals in men in the present subgroup analyses, with height providing the clearest representative pattern. In contrast, several metabolic indicators in men showed comparatively smaller coefficients, such as the high-glucose subgroup (β = 0.013) and the high-LDL-C subgroup (β = 0.028). Consistent with the interaction tests, the age-height interaction remained significant in males after BH-FDR correction, indicating a steeper age-related increase in CAVI among shorter men (Supplementary Table S1).

In women, the subgroup pattern differed, with relatively larger age–CAVI coefficients observed in several metabolic-related indicators and in some inflammatory or glycemic subgroups. Anthropometric subgroup coefficients in women were generally modest, whereas several metabolic and inflammatory subgroups showed relatively steeper age–CAVI associations. Neither BMI (interaction *p* = 0.8759) nor waist circumference (interaction *p* = 0.7384) emerged as a major age-interaction signal in relation to CAVI. Notably, steeper age–CAVI associations were observed in women with high HbA1c (β = 0.054; n = 11) and high CRP (β = 0.053), as indicated by the deep red blocks; the coefficient for the female high-HbA1c subgroup should be interpreted with caution given the small sample size, and the CRP-related contrast should also be interpreted cautiously because the corresponding overall interaction was not retained after additional adjustment. Larger coefficients were also observed in several lipid-related subgroups, including the high-LDL-C subgroup, although the corresponding LDL-C interaction should be interpreted cautiously in light of the updated overall analyses.

## 4. Discussion

This study investigated the age- and sex-specific correlates of arterial stiffness, as assessed by the cardio–ankle vascular index (CAVI), in a rigorously screened cohort of healthy Chinese adults. Several descriptive findings may be noted. First, CAVI showed a clear age-related increase in both sexes, with a steeper age–CAVI association after midlife [17,26,34]. Second, the associations between CAVI and factors from the anthropometric, metabolic, and inflammatory domains showed heterogeneous patterns, with the clearest overall interaction signals concentrated in a limited number of indicators. Third, sex-stratified analyses revealed distinct domain-specific association patterns, suggesting that subgroup association patterns related to vascular stiffness may differ between men and women [33,35,36,37]. Taken together, these results provide additional descriptive evidence on age- and sex-related association patterns of CAVI in apparently healthy adults and may support a more cautious age- and sex-sensitive interpretation of CAVI in preventive health settings, particularly with respect to height- and TG-related heterogeneity in the overall analyses [9,10,11,18].

### 4.1. Age-Related Patterns of CAVI and Implications for Early Vascular Aging

Our results are consistent with prior evidence that age is a major correlate of arterial stiffness even among healthy adults [8,17,26,38]. CAVI values increased progressively across age groups, with a steeper age–CAVI association beginning in participants over 50 years [10,18,34]. This pattern supports the concept of Early Vascular Aging (EVA), which suggests that vascular changes may begin earlier than expected based on metrical age alone [12]. In our cohort, some individuals in their 40s already exhibited CAVI levels approaching those observed in later decades, indicating that early vascular deviations may occur even in the absence of overt metabolic disease or hypertension. Establishing these age-specific patterns in a healthy East Asian population helps address an important reference gap, as descriptive data for CAVI have been largely derived from Western cohorts [15,26]. Clinically, these age-related patterns may help clinicians to distinguish between physiological age-related changes and potentially pathological elevations that may warrant further cardiometabolic evaluation.

### 4.2. Sex Differences in Arterial Stiffness and Possible Biological Correlates

Beyond age, our findings suggest sex-related differences in the age–CAVI association and in the subgroup patterns observed across physiological indicators. Men exhibited higher CAVI values overall and showed a steeper age-associated increase than women in the present sample; however, this descriptive pattern should not be interpreted as direct evidence of a faster progression of structural vascular aging [34,35,36]. Women demonstrated a distinct pattern: while their overall CAVI levels were lower, a noticeable midlife rise was observed, a pattern that may be compatible with midlife physiological changes. This pattern may be compatible with prior evidence linking midlife hormonal changes to vascular and metabolic alterations [33,35,39]. Our sex-stratified subgroup analyses suggest that the association patterns linked to vascular stiffness may differ by sex [33,36].

In men, relatively larger coefficients were more often observed within the anthropometric domain, particularly for height, which may be compatible with a more evident anthropometric-related descriptive pattern [31,40]. Higher body mass and related body-size characteristics can be accompanied by increased arterial wall stress and pulsatile load, although in the present analysis, the clearest anthropometric interaction signal was observed for height rather than for BMI or waist circumference [19]. In addition, shorter stature has been associated with arterial stiffness and steeper age–CAVI associations, providing a plausible context for the stronger height-related signal observed in men [31,40]. The concurrent signal for glycemic indicators in men was less consistent in the overall analyses and should therefore be interpreted cautiously despite its appearance in some subgroup displays [41]. In women, several metabolic-related strata, including TG and to a lesser extent LDL-C, showed comparatively larger subgroup coefficients, whereas the CRP-related contrast was not retained in the updated overall analyses [24,25,29,30,31]. One of the steepest age–CAVI associations in women was observed in the high-HbA1c stratum; however, this subgroup was small (n = 11; Table 10), and the estimate should be interpreted cautiously. These findings suggest that vascular aging is not uniform across sexes but may reflect sex-specific physiological contexts that may vary over the lifespan [35,36,37].

### 4.3. Relative Association Patterns Across the Three Biological Domains

A key objective of this study was to examine whether factors from the three biological domains defined in the present analytic framework, anthropometric, metabolic, and inflammatory, showed different subgroup association patterns with arterial stiffness. Our findings show domain-specific contribution patterns, while recognizing that the overall interaction evidence was concentrated in only a limited number of indicators.

Anthropometric domain: Height displayed a consistent inverse association with CAVI [26,28,41]. Physiologically, this may be related to the length of the arterial tree; taller individuals have longer arterial paths, which may delay the return of reflected pressure waves, which could theoretically be associated with lower arterial stiffness readings [15,26,28]. This signal was particularly evident in men in the present subgroup analyses, although the clearest anthropometric interaction signal was observed for height, while BMI and waist circumference did not emerge as major interaction signals.

Metabolic domain: Lipid-related factors exhibited heterogeneous associations with CAVI, with triglycerides showing the clearest pattern, whereas LDL-C showed a weaker pattern after additional adjustment [5,19,20,29,30,31]. Elevated TG was consistently associated with a steeper age–CAVI association, whereas the corresponding LDL-C pattern was limited [5,19,20,29,30,31]. These findings are consistent with the relevance of lipid-related indicators to age-related variation in CAVI, in line with the prior literature involving oxidative stress, endothelial dysfunction, and smooth muscle remodeling [19,27,42]. Notably, some of these signals appeared more evident in women in the present subgroup analyses; however, these descriptive sex-specific patterns should be interpreted cautiously and not as definitive evidence of a stronger metabolic influence on female vascular aging, with TG providing the clearest corresponding signal and LDL-C representing a secondary pattern [25,35,36].

Inflammatory domain: CRP showed an initial interaction signal in the earlier models, but this signal was attenuated after adjustment for blood pressure and was not retained in the overall analyses [24,25]. Additionally, CRP-related subgroup differences appeared more evident in women; however, this pattern should be interpreted cautiously [20,21,29].

Taken together, these patterns suggest that the association patterns related to arterial stiffness are heterogeneous [3,5,37,40]. Although the cross-sectional design precludes causal inference, the observed subgroup patterns suggest that signals from the anthropometric domain may be more evident in some male subgroups, whereas signals from the metabolic domains may be more evident in some female subgroups, with inflammatory contrasts remaining descriptive and less stable in the overall analyses [24,25,29,30,31,35,36,37].

### 4.4. Clinical Implications

This study has several potential implications for clinical practice. First, the age- and sex-specific CAVI patterns observed in this rigorously screened cohort may offer descriptive reference information for interpreting CAVI values in apparently healthy East Asian adults [13,18,32,39]. Importantly, the present findings should be interpreted as pertaining primarily to large-vessel stiffness assessed by CAVI and should not be directly extrapolated to resistance (small) arterial stiffness or microvascular dysfunction. Deviations from these descriptive trajectories may help flag individuals at risk of EVA despite otherwise unremarkable cardiometabolic profiles [33]. Second, our domain-based findings may inform hypothesis generation regarding sex-specific risk interpretation in preventive health assessment, with the clearest overall signals observed for height and TG. If confirmed in future studies, anthropometric indicators, particularly height, may warrant particular attention when interpreting subgroup differences in men. For women, metabolic indicators, particularly TG, may warrant particular attention in some female subgroups [24,25,29,30,31,35,36]. Finally, recognizing that arterial stiffness is shaped by the interplay of multiple biological domains supports the use of CAVI as part of a comprehensive cardiovascular risk stratification approach rather than a standalone indicator [8,37,38].

### 4.5. Limitations

This study has several limitations. First, its cross-sectional design precludes causal inference. Second, although the overall analytic sample included 525 participants, sample sizes in several subgroups became limited after sex stratification and biomarker-based subgrouping; therefore, some subgroup-specific estimates should be interpreted cautiously. Third, the present study focused on CAVI and did not include the heart–thigh beta index (htβ), so comparison with a more central arterial stiffness measure was not possible. Fourth, the menopausal status was not available in the present dataset; therefore, the transient midlife elevation observed among women should be interpreted cautiously rather than as direct evidence of a menopausal mechanism. Future studies with larger samples, longitudinal follow-up, more comprehensive covariate adjustment, and, where possible, joint assessment of CAVI and htβ are warranted.

## 5. Conclusions

In conclusion, in this cohort of apparently healthy Chinese adults, we found that arterial stiffness assessed by CAVI rose steadily with age and seemed to climb more steeply after the fifth decade onward. Men had higher CAVI values and a slightly faster age-related increase than women, whereas women showed a transient midlife elevation in CAVI. In subgroup interaction analyses, anthropometric signals appeared more prominent in men, with height providing the clearest representative pattern, whereas metabolic signals appeared more prominent in women, with TG providing the clearest corresponding signal.

## Figures and Tables

**Table 1:** Mean ± standard deviation of Cardio–Ankle Vascular Index (CAVI) by age group.

Age Group	Total		Male		Female	
	n	Mean ± SD	n	Mean ± SD	n	Mean ± SD
1	50	5.85 ± 0.38	22	5.92 ± 0.31	28	5.80 ± 0.42
2	174	6.00 ± 0.41	84	6.08 ± 0.48	90	5.92 ± 0.31
3	162	6.09 ± 0.45	82	6.07 ± 0.38	80	6.11 ± 0.52
4	104	6.34 ± 0.80 ^abc^	53	6.46 ± 0.85 ^abc^	51	6.21 ± 0.73 ^ab^
5	35	6.88 ± 1.32 ^abcd^	24	7.01 ± 1.35 ^abcd^	11	6.59 ± 1.27 ^ab^

Note: a: *p* < 0.05 compared to 1; b: *p* < 0.05 compared to 2; c: *p* < 0.05 compared to 3; d: *p* < 0.05 compared to 4.

**Table 2:** Regression analysis of the effect of age on Arterial Stiffness (CAVI).

Variant	Model 1 β (95%CI) *p*	Model 2 β (95%CI) *p*
(CAVI)	0.023 (0.018, 0.028) <0.00001	0.023 (0.018, 0.027) <0.00001

Note: Dependent variable: cardio–ankle vascular index (CAVI), independent variable: age. Model 1: unadjusted variable, Model 2: adjusted variable adjusted for sex.

**Table 3:** Sex-stratified association between age and arterial stiffness (CAVI).

Variant	Male β (95%CI) *p*	Female β (95%CI) *p*
(CAVI)	0.026 (0.019, 0.033) <0.0001	0.019 (0.012, 0.026) <0.0001

Note: Dependent variable: cardio–ankle vascular index (CAVI); independent variable: age; effect modifier: sex.

**Table 4:** Stratified regression and interaction test of the influencing factors (anthropometric indicators).

Indicator Stratification	n	Model 1 β (95%CI) *p*	*p*	Model 2 β (95%CI) *p*	*p*	Model 3 β (95%CI) *p*	*p*	Model 4 β (95%CI) *p*	*p*
Height	525								
high	270	0.015 (0.007, 0.023) <0.0001	0.0051	0.016 (0.008, 0.024) <0.0001	0.0154	0.016 (0.009, 0.024) <0.0001	0.0157	0.017 (0.009, 0.024) <0.00001	0.0104
low	255	0.030 (0.023, 0.037) <0.0001		0.029 (0.022, 0.036) <0.0001		0.028 (0.021, 0.035) <0.0001		0.029 (0.021, 0.036) <0.00001	
Weight	525								
high	257	0.028 (0.021, 0.036) <0.0001	0.1648	0.029 (0.021, 0.037) <0.0001	0.0680	0.024 (0.017, 0.031) <0.0001	0.2170	0.024 (0.017, 0.031) <0.00001	0.2316
low	268	0.021 (0.015, 0.028) <0.0001		0.020 (0.013, 0.026) <0.0001		0.018 (0.011, 0.026) <0.0001		0.018 (0.011, 0.026) <0.00001	
BMI	525								
high	229	0.028 (0.021, 0.036) <0.0001	0.0952	0.028 (0.020, 0.036) <0.0001	0.0830	0.026 (0.018, 0.033) <0.0001	0.1469	0.026 (0.018, 0.033) <0.00001	0.1335
low	296	0.020 (0.013, 0.026) <0.0001		0.019 (0.012, 0.025) <0.0001		0.017 (0.011, 0.024) <0.0001		0.017 (0.010, 0.024) <0.00001	
Waist circumference	492								
high	253	0.022 (0.015, 0.030) <0.0001	0.7246	0.022 (0.015, 0.030) <0.0001	0.7744	0.022 (0.015, 0.029) <0.0001	0.8566	0.022 (0.015, 0.029) <0.00001	0.8535
low	239	0.024 (0.017, 0.032) <0.0001		0.022 (0.014, 0.030) <0.0001		0.019 (0.011, 0.027) <0.0001		0.019 (0.011, 0.027) <0.00001	
Hip measurement	492								
high	243	0.027 (0.019, 0.035) <0.0001	0.4279	0.027 (0.019, 0.035) <0.0001	0.3400	0.024 (0.016, 0.031) <0.0001	0.5619	0.024 (0.016, 0.032) <0.00001	0.4538
low	249	0.023 (0.015, 0.030) <0.0001		0.022 (0.014, 0.029) <0.0001		0.021 (0.013, 0.029) <0.0001		0.020 (0.012, 0.028) <0.00001	
Pulse rate	525								
high	268	0.021 (0.014, 0.028) <0.0001	0.3294	0.020 (0.013, 0.027) <0.0001	0.2334	0.018 (0.012, 0.024) <0.0001	0.1230	0.019 (0.013, 0.025) <0.00001	0.4624
low	257	0.026 (0.019, 0.033) <0.0001		0.026 (0.019, 0.033) <0.0001		0.025 (0.017, 0.034) <0.0001		0.024 (0.016, 0.032) <0.00001	

Note: Dependent variable: Cardio-ankle vascular index (CAVI); independent variable: age, and the data in the table are expressed as β (95% CI) *p*. Model 1: no adjustment variable, Model 2: sex as adjustment variable, Model 3: sex, Systolic blood pressure (SBP) and Diastolic blood pressure (DBP) as adjustment variable, Model 4: additionally adjusted for smoking status.

**Table 5:** Mean ± standard deviation of anthropometric indicators by age group.

	Height	Weight	BMI	Waist Circumference	Hip Measurement	Pulse Rate
1	169.9 ± 9.74	83.7 ± 21.0	29.02 ± 5.17	94.9 ± 14.7	108.1 ± 10.9	83.9 ± 9.39
2	168.7 ± 9.83	78.1 ± 19.5	27.40 ± 5.12	92.4 ± 14.4	102.8 ± 9.72 ^a^	78.6 ± 9.83 ^a^
3	167.0 ± 8.08	73.6 ± 14.6 ^a^	26.30 ± 4.81 ^a^	90.9 ± 12.7	100.0 ± 7.35 ^ab^	78.9 ± 10.0 ^a^
4	164.9 ± 7.70 ^ab^	70.3 ± 12.7 ^ab^	25.79 ± 4.90 ^a^	88.9 ± 13.1 ^a^	98.7 ± 7.18 ^ab^	75.1 ± 10.3 ^abc^
5	164.3 ± 6.07 ^ab^	69.4 ± 10.4 ^ab^	25.45 ± 5.28 ^a^	90.9 ± 13.1	98.4 ± 7.38 ^ab^	74.2 ± 10.3 ^a^

Note: BMI, body mass index. a: *p* < 0.05 compared to 1; b: *p* < 0.05 compared to 2; c: *p* < 0.05 compared to 3.

**Table 6:** Stratified regression and interaction tests for the influences (lipid indicators).

Indicator Stratification	n	Model 1 β (95%CI) *p*	*p*	Model 2 β (95%CI) *p*	*p*	Model 3 β (95%CI) *p*	*p*	Model 4 β (95%CI) *p*	*p*
LDL-C	514								
high	204	0.030 (0.023, 0.038) <0.0001	0.0076	0.030 (0.023, 0.038) <0.0001	0.0056	0.029 (0.020, 0.037) <0.0001	0.0167	0.029 (0.020, 0.037) <0.00001	0.0157
low	310	0.017 (0.010, 0.023) <0.0001		0.016 (0.010, 0.023) <0.0001		0.015 (0.009, 0.022) <0.0001		0.015 (0.009, 0.021) <0.00001	
TG	514								
high	257	0.031 (0.024, 0.038) <0.0001	0.0010	0.031 (0.024, 0.038) <0.0001	0.0008	0.030 (0.022, 0.038) <0.0001	<0.0001	0.030 (0.022, 0.038) <0.00001	<0.0001
low	257	0.015 (0.008, 0.021) <0.0001		0.014 (0.007, 0.021) <0.0001		0.013 (0.007, 0.018) <0.0001		0.012 (0.007, 0.018) <0.0001	
TC	514								
high	182	0.025 (0.017, 0.033) <0.0001	0.6122	0.026 (0.017, 0.034) <0.0001	0.4030	0.027 (0.018, 0.036) <0.0001	0.2023	0.027 (0.018, 0.036) <0.00001	0.2140
low	332	0.022 (0.016, 0.029) <0.0001		0.021 (0.015, 0.028) <0.0001		0.018 (0.012, 0.024) <0.0001		0.018 (0.012, 0.024) <0.00001	
HDL-C	514								
high	321	0.027 (0.021, 0.033) <0.0001	0.0164	0.027 (0.021, 0.033) <0.0001	0.0216	0.024 (0.017, 0.031) <0.0001	0.0583	0.024 (0.017, 0.031) <0.00001	0.0594
low	193	0.014 (0.005, 0.023) 0.0018		0.014 (0.005, 0.023) 0.0018		0.015 (0.009, 0.020) <0.0001		0.014 (0.009, 0.020) <0.00001	

Note: CAVI, cardio-ankle vascular index; LDL-C, low-density lipoprotein cholesterol; TG, triglyceride; TC, total cholesterol; HDL-C, high-density lipoprotein cholesterol; SBP, systolic blood pressure; DBP, diastolic blood pressure. CAVI is the dependent variable, and age is the independent variable; data in the table are expressed as β (95% CI) and *p* value. Model 1: no adjustment variable, Model 2: sex as adjustment variable, Model 3: sex, SBP and DBP as adjustment variables, Model 4: additionally adjusted for smoking status.

**Table 7:** Mean ± standard deviation of lipid indices by age group.

	LDL-C	TG	TC	HDL-C
1	1.27 ± 0.59	1.27 ± 0.71	4.62 ± 0.91	1.25 ± 0.27
2	1.34 ± 0.69	1.77 ± 2.04	4.87 ± 0.88	1.26 ± 0.32
3	1.51 ± 0.66	1.86 ± 1.38	4.93 ± 0.94	1.29 ± 0.38
4	1.57 ± 0.84	1.75 ± 1.11	5.04 ± 0.93 ^a^	1.38 ± 0.34 ^b^
5	1.67 ± 0.77 ^a^	1.75 ± 1.22	5.23 ± 0.97 ^a^	1.32 ± 0.28

Note: a: *p* < 0.05 compared to 1; b: *p* < 0.05 compared to 2.

**Table 8:** Stratified regression and interaction tests for glycemic and inflammatory indicators.

Indicator Stratification	n	Model 1 β (95%CI) *p*	*p*	Model 2 β (95%CI) *p*	*p*	Model 3 β (95%CI) *p*	*p*	Model 4 β (95%CI) *p*	*p*
HbA1c	514								
high	184	0.027 (0.019, 0.034) <0.0001	0.1991	0.027 (0.019, 0.034) <0.0001	0.1969	0.024 (0.015, 0.033) <0.0001	0.2574	0.024 (0.015, 0.033) <0.00001	0.2831
low	330	0.020 (0.013, 0.026) <0.0001		0.020 (0.013, 0.026) <0.0001		0.019 (0.013, 0.025) <0.0001		0.019 (0.013, 0.025) <0.00001	
GLU	514								
high	110	0.021 (0.011, 0.031) <0.0001	0.7663	0.021 (0.011, 0.031) <0.0001	0.7834	0.021 (0.009, 0.032) 0.0006	0.9637	0.021 (0.009, 0.033) 0.0006	0.9996
low	404	0.023 (0.017, 0.029) <0.0001		0.023 (0.017, 0.028) <0.0001		0.021 (0.015, 0.027) <0.0001		0.020 (0.015, 0.026) <0.00001	
CRP	514								
high	52	0.043 (0.028, 0.058) <0.0001	0.0091	0.042 (0.027, 0.057) <0.0001	0.0081	0.033 (0.018, 0.048) <0.0001	0.1156	0.033 (0.018, 0.049) <0.0001	0.1210
low	462	0.022 (0.016, 0.027) <0.0001		0.021 (0.016, 0.026) <0.0001		0.020 (0.015, 0.026) <0.0001		0.020 (0.015, 0.025) <0.00001	

Note: CAVI, cardio-ankle vascular index; HbA1c, glycated hemoglobin; GLU, fasting glucose; CRP, C-reactive protein; SBP, systolic blood pressure; DBP, diastolic blood pressure. CAVI is the dependent variable, age is the independent variable, and the data in the table are expressed as β (95% CI) and *p* value. Model 1: no adjustment variable, Model 2: sex as adjustment variable, Model 3: sex, SBP and DBP as adjustment variables, Model 4: additionally adjusted for smoking status.

**Table 9:** Mean ± standard deviation of glycemic and inflammatory indicators by age group.

	HbA1c	GLU	CRP
1	5.06 ± 0.37	5.14 ± 0.70	3.29 ± 2.51
2	5.08 ± 0.55	5.50 ± 1.11	2.88 ± 2.59
3	5.28 ± 0.64 ^b^	5.68 ± 1.19	2.32 ± 2.10 ^a^
4	5.40 ± 0.84 ^ab^	5.99 ± 1.87 ^ab^	2.34 ± 1.77
5	5.67 ± 0.68 ^abc^	6.51 ± 1.56 ^abc^	2.69 ± 2.58

Note: a: *p* < 0.05 compared to 1; b: *p* < 0.05 compared to 2; c: *p* < 0.05 compared to 3.

**Table 10:** Stratified regression of influences on interaction tests (male and female).

	Male				Female			
Indicator Stratification	Sex-Specific Subgroup Range	n	Model 1 β (95%CI) *p*	*p*	Sex-Specific Subgroup Range	n	Model 1 β (95%CI) *p*	*p*
Pulse rate		262				260		
high	79–103	118	0.027 (0.016, 0.038) <0.0001	0.1537	80–99	132	0.023 (0.013, 0.033) <0.0001	0.3691
low	50–78	144	0.016 (0.006, 0.026) 0.0015		52–79	128	0.017 (0.008, 0.026) 0.0002	
height		265				260		
high	173.5–195	136	0.011 (−0.002, 0.024) 0.0977	0.0028	161–179.5	134	0.019 (0.010, 0.028) <0.0001	0.9820
low	157–173	129	0.037 (0.026, 0.047) <0.0001		144.5–160.5	126	0.019 (0.010, 0.028) <0.0001	
weight		265				260		
high	82.1–137.1	130	0.036 (0.024, 0.048) <0.0001	0.1342	64–136.5	127	0.023 (0.014, 0.033) <0.0001	0.2356
low	55.1–81.9	135	0.024 (0.013, 0.034) <0.0001		43.6–63.9	133	0.016 (0.007, 0.024) 0.0006	
BMI		265		0.9533		260		0.8759
high	28.12–45.83	132	0.023 (0.014, 0.031) <0.0001		25.47–39.07	130	0.014 (0.005, 0.023) 0.0025	
low	15.31–28.07	133	0.023 (0.011, 0.035) 0.0003		14.80–25.43	130	0.015 (0.006, 0.025) 0.0021	
Waist circumference		265		0.4629		260		0.7384
high	90.2–140.0	132	0.025 (0.017, 0.034) <0.0001		90.5–134.6	130	0.014 (0.005, 0.024) 0.0034	
low	59.9–90.1	133	0.020 (0.008, 0.032) 0.0018		60.0–90.3	130	0.017 (0.007, 0.026) 0.0004	
LDL-C		263				251		
high	1.79–3.78	171	0.028 (0.019, 0.037) <0.0001	0.4538	2.08–3.56	33	0.042 (0.026, 0.057) <0.0001	0.0014
low	0.32–1.74	92	0.022 (0.009, 0.035) 0.0010		0.04–2.05	218	0.014 (0.007, 0.021) 0.0002	
TG		263				251		
high	1.16–21.7	201	0.029 (0.020, 0.038) <0.0001	0.2278	1.56–8.31	56	0.039 (0.026, 0.053) <0.0001	0.0006
low	0.39–1.15	62	0.019 (0.005, 0.033) 0.0069		0.06–1.54	196	0.012 (0.005, 0.019) 0.0015	
TC		263				251		
high	5.19–8.51	103	0.029 (0.017, 0.040) <0.0001	0.5394	5.09–8.19	79	0.021 (0.009, 0.033) 0.0005	0.6765
low	2.21–5.18	160	0.024 (0.015, 0.034) <0.0001		2.31–5.08	172	0.018 (0.010, 0.027) <0.0001	
HbA1c		263				251		
high	5.1–10.0	173	0.024 (0.016, 0.033) <0.0001	0.2862	5.7–8.1	11	0.054 (0.026, 0.082) 0.0002	0.0089
low	4.3–5	90	0.033 (0.019, 0.047) <0.0001		4–5.6	240	0.015 (0.009, 0.022) <0.0001	
GLU		263				251		
high	6.11–18.24	71	0.013 (−0.000, 0.025) 0.0539	0.0100	6.12–15.43	39	0.043 (0.026, 0.059) <0.0001	0.0012
low	3.94–6.1	192	0.033 (0.024, 0.042) <0.0001		3.82–6.1	212	0.013 (0.006, 0.020) 0.0006	
HDL-C		263				251		
high	1.3–2.14	171	0.030 (0.021, 0.039) <0.0001	0.0412	1.3–2.94	150	0.021 (0.013, 0.030) <0.0001	0.3764
low	0.52–1.29	92	0.013 (−0.000, 0.027) 0.0583		0.68–1.29	101	0.015 (0.004, 0.026) 0.0082	
TRL-C		263				251		
high	0.57–4.88	185	0.029 (0.020, 0.038) <0.0001	0.3090	0.57–5	105	0.023 (0.013, 0.033) <0.0001	0.2144
low	0.02–0.56	78	0.021 (0.009, 0.033) 0.0011		0.17–0.56	146	0.015 (0.006, 0.024) 0.0013	
CRP								
high	5.04–16.57	26	0.029 (0.004, 0.055) 0.0250	0.8045	5.04–16.57	26	0.053 (0.036, 0.070) <0.0001	<0.0001
low	0.25–4.96	237	0.026 (0.018, 0.034) <0.0001		0.25–4.96	225	0.015 (0.008, 0.021) <0.0001	

Note: CAVI, cardio-ankle vascular index; BMI, body mass index; LDL-C, low-density lipoprotein cholesterol; TG, triglyceride; TC, total cholesterol; HbA1c, glycated hemoglobin; GLU, fasting glucose; HDL-C, high-density lipoprotein cholesterol; TRL-C, triglyceride-rich lipoprotein cholesterol; CRP, C-reactive protein. Dependent variable: CAVI; independent variable: age. Data are expressed as β (95% CI) and *p* value from sex-stratified linear regression analyses. The separate *p* column indicates the interaction *p* value for the difference in age–CAVI slopes between high and low subgroups within each sex. Model 1 was unadjusted. Sex-specific subgroup ranges were applied separately in men and women.

## Data Availability

The data that support the findings of this study are available from the corresponding author upon reasonable request.

## Supplementary Materials

The following supporting information can be downloaded at: https://www.mdpi.com/article/10.3390/metabo16050300/s1, Table S1: Interaction tests with BH-FDR adjustment; Table S2: CRP Sensitivity Analysis: Exclude Participants with CRP > 10 mg/L; Table S3: CRP Sensitivity Analysis: CRP distribution before and after exclusion; Table S4: Cut-off values used for subgroup definitions.

## Abbreviations

EVA: early vascular aging; CAVI: cardio–ankle vascular index; BMI: body mass index; CRP: C-reactive protein; HbA1c: glycated hemoglobin; GLU: fasting glucose; HDL-C: high-density lipoprotein cholesterol; LDL-C: low-density lipoprotein cholesterol; TC: total cholesterol; PWV: pulse wave velocity; TG: triglyceride; TRL-C: triglyceride-rich lipoprotein cholesterol; SBP: systolic blood pressure; DBP: diastolic blood pressure; FDR: false discovery rate; BH: Benjamini–Hochberg; HSD: honestly significant difference; SOP: standard operating procedure.

## References

[1] Vlachopoulos C., Aznaouridis K., Stefanadis C. (2010). Prediction of cardiovascular events and all-cause mortality with arterial stiffness: A systematic review and meta-analysis. J. Am. Coll. Cardiol..

[2] Boutouyrie P., Chowienczyk P., Humphrey J.D., Mitchell G.F. (2021). Arterial stiffness and cardiovascular risk in hypertension. Circ. Res..

[3] Regnault V., Lacolley P., Laurent S. (2024). Arterial stiffness: From basic primers to integrative physiology. Annu. Rev. Physiol..

[4] Lakatta E.G., Levy D. (2003). Arterial and cardiac aging: Major shareholders in cardiovascular disease enterprises: Part I: Aging arteries: A ‘set up’ for vascular disease. Circulation.

[5] Zieman S.J., Melenovsky V., Kass D.A. (2005). Mechanisms, pathophysiology, and therapy of arterial stiffness. Arterioscler. Thromb. Vasc. Biol..

[6] Laurent S., Boutouyrie P. (2015). The structural factor of hypertension: Large and small artery alterations. Circ. Res..

[7] Chen W., Li S., Fernandez C., Sun D., Lai C.-C., Zhang T., Bazzano L., Urbina E.M., Deng H.-W. (2016). Temporal relationship between elevated blood pressure and arterial stiffening among middle-aged Black and White adults: The Bogalusa Heart Study. Am. J. Epidemiol..

[8] Cecelja M., Chowienczyk P. (2012). Role of arterial stiffness in cardiovascular disease. JRSM Cardiovasc. Dis..

[9] Yambe T., Yoshizawa M., Saijo Y., Yamaguchi T., Shibata M., Konno S., Nitta S., Kuwayama T. (2004). Brachio-ankle pulse wave velocity and cardio-ankle vascular index (CAVI). Biomed. Pharmacother..

[10] Namekata T., Suzuki K., Ishizuka N., Shirai K. (2011). Establishing baseline criteria of cardio-ankle vascular index as a new indicator of arteriosclerosis: A cross-sectional study. BMC Cardiovasc. Disord..

[11] Shirai K., Hiruta N., Song M., Kurosu T., Suzuki J., Tomaru T., Miyashita Y., Saiki A., Takahashi M., Suzuki K. (2011). Cardio-ankle vascular index (CAVI) as a novel indicator of arterial stiffness: Theory, evidence and perspectives. J. Atheroscler. Thromb..

[12] Nilsson P.M., Boutouyrie P., Cunha P., Kotsis V., Narkiewicz K., Parati G., Rietzschel E., Scuteri A., Laurent S. (2013). Early vascular ageing in translation: From laboratory investigations to clinical applications in cardiovascular prevention. J. Hypertens..

[13] Li S., Chen W., Srinivasan S.R., Berenson G.S. (2005). Influence of metabolic syndrome on arterial stiffness and its age-related change in young adults: The Bogalusa Heart Study. Atherosclerosis.

[14] Aatola H., Hutri-Kähönen N., Juonala M., Viikari J.S.A., Hulkkonen J., Laitinen T., Taittonen L., Lehtimäki T., Raitakari O.T., Kähönen M. (2010). Lifetime risk factors and arterial pulse wave velocity in adulthood: The Cardiovascular Risk in Young Finns Study. Hypertension.

[15] Hickson S.S., Butlin M., Graves M., Taviani V., Avolio A.P., McEniery C.M., Wilkinson I.B. (2010). The relationship of age with regional aortic stiffness and diameter. JACC Cardiovasc. Imaging.

[16] Laurent S., Boutouyrie P. (2007). Arterial stiffness: A new surrogate end point for cardiovascular disease?. J. Nephrol..

[17] Sun Z. (2015). Aging, arterial stiffness, and hypertension. Hypertension.

[18] Kubozono T., Miyata M., Ueyama K., Nagaki A., Otsuji Y., Kusano K., Kubozono O., Tei C. (2007). Clinical significance and reproducibility of new arterial distensibility index. Circ. J..

[19] Mäki-Petäjä K.M., Wilkinson I.B. (2012). Inflammation and large arteries: Potential mechanisms for inflammation-induced arterial stiffness. Artery Res..

[20] deGoma E.M., deGoma R.L., Rader D.J. (2008). Beyond high-density lipoprotein cholesterol levels: Evaluating high-density lipoprotein function as influenced by novel therapeutic approaches. J. Am. Coll. Cardiol..

[21] Jia G., Aroor A.R., Hill M.A., Sowers J.R. (2018). Role of renin-angiotensin-aldosterone system activation in promoting cardiovascular fibrosis and stiffness. Hypertension.

[22] Wu M.Y., Li C.J., Hou M.F., Chu P.Y. (2017). New insights into the role of inflammation in the pathogenesis of atherosclerosis. Int. J. Mol. Sci..

[23] Libby P., Ridker P.M., Hansson G.K. (2011). Progress and challenges in translating the biology of atherosclerosis. Nature.

[24] Yasmin, McEniery C.M., Wallace S., Mackenzie I.S., Cockcroft J.R., Wilkinson I.B. (2004). C-reactive protein is associated with arterial stiffness in apparently healthy individuals. Arterioscler. Thromb. Vasc. Biol..

[25] Gómez-Marcos M.A., Recio-Rodríguez J.I., Patiño-Alonso M.C., Agudo-Conde C., Gómez-Sanchez L., Rodríguez-Sanchez E., Gómez-Sanchez M., Martínez-Vizcaíno V., García-Ortiz L. (2012). Relationships between high-sensitive C-reactive protein and markers of arterial stiffness in hypertensive patients: Differences by sex. BMC Cardiovasc. Disord..

[26] (2010). The Reference Values for Arterial Stiffness’ Collaboration. Determinants of pulse wave velocity in healthy people and in the presence of cardiovascular risk factors: ‘Establishing normal and reference values’. Eur. Heart J..

[27] Gimbrone M.A., García-Cardeña G. (2016). Endothelial cell dysfunction and the pathobiology of atherosclerosis. Circ. Res..

[28] Koivistoinen T., Kööbi T., Jula A., Hutri-Kähönen N., Raitakari O.T., Majahalme S., Kukkonen-Harjula K., Lehtimäki T., Reunanen A., Viikari J. (2007). Pulse wave velocity reference values in healthy adults aged 26–75 years. Clin. Physiol. Funct. Imaging.

[29] Si X.B., Liu W. (2019). Relationship between blood lipid and arterial stiffness in hypertension. Clin. Investig. Med..

[30] Nagayama D., Watanabe Y., Saiki A., Shirai K., Tatsuno I. (2018). Lipid parameters are independently associated with cardio-ankle vascular index (CAVI) in healthy Japanese subjects. J. Atheroscler. Thromb..

[31] Wang L., Zhi F., Gao B., Ni J., Liu Y., Mo X., Huang J. (2020). Association between lipid profiles and arterial stiffness: A secondary analysis based on a cross-sectional study. J. Int. Med. Res..

[32] Mitchell G.F., Vita J.A., Larson M.G., Parise H., Keyes M.J., Warner E., Vasan R.S., Levy D., Benjamin E.J. (2005). Cross-sectional relations of peripheral microvascular function, cardiovascular disease risk factors, and aortic stiffness: The Framingham Heart Study. Circulation.

[33] Stanhewicz A.E., Wenner M.M., Stachenfeld N.S. (2018). Sex differences in endothelial function important to vascular health and overall cardiovascular disease risk across the lifespan. Am. J. Physiol. Heart Circ. Physiol..

[34] Tomiyama H., Yamashina A., Arai T., Hirose K., Koji Y., Chikamori T., Hori S., Yamamoto Y., Doba N., Hinohara S. (2003). Influences of age and gender on results of noninvasive brachial-ankle pulse wave velocity measurement: A survey of 12,517 subjects. Atherosclerosis.

[35] Moreau K.L., Hildreth K.L. (2014). Vascular aging across the menopause transition in healthy women. Adv. Vasc. Med..

[36] Robert J. (2023). Sex differences in vascular endothelial cells. Atherosclerosis.

[37] Herzog M.J., Müller P., Lechner K., Stiebler M., Arndt P., Kunz M., Ahrens D., Schmeißer A., Schreiber S., Braun-Dullaeus R.C. (2025). Arterial stiffness and vascular aging: Mechanisms, prevention, and therapy. Signal Transduct. Target. Ther..

[38] Ben-Shlomo Y., Spears M., Boustred C., May M., Anderson S.G., Benjamin E.J., Boutouyrie P., Cameron J., Chen C.H., Cruickshank J.K. (2014). Aortic pulse wave velocity improves cardiovascular event prediction: An individual participant meta-analysis of prospective observational data from 17,635 subjects. J. Am. Coll. Cardiol..

[39] Miller V.M., Duckles S.P. (2008). Vascular actions of estrogens: Functional implications. Pharmacol. Rev..

[40] Laurent S., Cockcroft J., Van Bortel L., Boutouyrie P., Giannattasio C., Hayoz D., Pannier B., Vlachopoulos C., Wilkinson I., Struijker-Boudier H. (2006). Expert consensus document on arterial stiffness: Methodological issues and clinical applications. Eur. Heart J..

[41] Sugawara J., Hayashi K., Yokoi T., Tanaka H. (2008). Age-associated elongation of the ascending aorta in adults. JACC Cardiovasc. Imaging.

[42] Harvey A., Montezano A.C., Lopes R.A., Rios F., Touyz R.M. (2016). Vascular fibrosis in aging and hypertension: Molecular mechanisms and clinical implications. Can. J. Cardiol..

