# Supplementary material for: Age- and Sex-Specific Patterns of Arterial Stiffness Assessed by Cardio–Ankle Vascular Index in Apparently Healthy Chinese Adults: A Cross-Sectional Study
Source: Metabolites. 2026 Apr 29;16(5):300. doi: 10.3390/metabo16050300 (PMC13208408; doi:10.3390/metabo16050300)
Supplement: Supplementary file 1 [file metabolites-16-00300-s001.zip › metabolites-4236319-supplementary.pdf]

Supplementary appendix

Age- and Sex-Specific Patterns of Arterial Stiffness Assessed by Cardio-Ankle Vascular Index in Apparently Healthy Chinese Adults: A Cross-Sectional Study

Supplementary Table S1. Interaction tests with BH-FDR adjustment

Comparison	z (approx.)	Unadjusted p	BH adjusted p	Significant after FDR
Age & Height (high vs low; Table 4; Model 1)	z=-2.801	0.0051	0.0387	Yes
Age & Height (high vs low; Table 4; Model 2)	z=-2.423	0.0154	0.0577	No
Age & Height (high vs low; Table 4; Model 3)	z=2.417	0.0157	0.0577	No
Age & Height (high vs low; Table 4; Model 4)	z=2.561	0.0104	0.0466	Yes
Age & Weight (high vs low; Table 4; Model 1)	z=1.389	0.1648	0.3385	No
Age & Weight (high vs low; Table 4; Model 2)	z=1.825	0.0680	0.1914	No
Age & Weight (high vs low; Table 4; Model 3)	z=1.235	0.2170	0.3810	No
Age & Weight (high vs low; Table 4; Model 4)	z=1.196	0.2316	0.3810	No
Age & BMI (high vs low; Table 4; Model 1)	z=1.668	0.0952	0.2495	No
Age & BMI (high vs low; Table 4; Model 2)	z=1.733	0.0830	0.2253	No
Age & BMI (high vs low; Table 4; Model 3)	z=1.451	0.1469	0.3190	No
Age & BMI (high vs low; Table 4; Model 4)	z=1.501	0.1335	0.3000	No
Age & Waist circumference (high vs low; Table 4; Model 1)	z=-0.352	0.7246	0.8472	No

Comparison	z (approx.)	Unadjusted p	BH adjusted p	Significant after FDR
Age & Waist circumference (high vs low; Table 4; Model 2)	z=0.287	0.7744	0.8756	No
Age & Waist circumference (high vs low; Table 4; Model 3)	z=0.181	0.8566	0.9169	No
Age & Waist circumference (high vs low; Table 4; Model 4)	z=0.185	0.8535	0.9169	No
Age & Hip measurement (high vs low; Table 4; Model 1)	z=0.793	0.4279	0.5705	No
Age & Hip measurement (high vs low; Table 4; Model 2)	z=0.954	0.3400	0.4875	No
Age & Hip measurement (high vs low; Table 4; Model 3)	z=0.580	0.5619	0.6888	No
Age & Hip measurement (high vs low; Table 4; Model 4)	z=0.749	0.4538	0.5846	No
Age & Pulse rate (high vs low; Table 4; Model 1)	z=-0.975	0.3294	0.4814	No
Age & Pulse rate (high vs low; Table 4; Model 2)	z=-1.192	0.2334	0.3810	No
Age & Pulse rate (high vs low; Table 4; Model 3)	z=-1.542	0.1230	0.2921	No
Age & Pulse rate (high vs low; Table 4; Model 4)	z=-0.735	0.4624	0.5857	No
Age & LDL-C (high vs low; Table 6; Model 1)	z=2.669	0.0076	0.0461	Yes
Age & LDL-C (high vs low; Table 6; Model 2)	z=2.770	0.0056	0.0387	Yes
Age & LDL-C (high vs low; Table 6; Model 3)	z=2.392	0.0167	0.0577	No
Age & LDL-C (high vs low; Table 6; Model 4)	z=2.417	0.0157	0.0577	No
Age & TG (high vs low; Table 6; Model 1)	z=3.291	0.0010	0.0127	Yes
Age & TG (high vs low; Table 6; Model 2)	z=3.353	0.0008	0.0122	Yes
Age & TG (high vs low; Table 6; Model 3)	z=3.959	<0.0001	0.0019	Yes

Comparison	z (approx.)	Unadjusted p	BH adjusted p	Significant after FDR
Age & TG (high vs low; Table 6; Model 4)	z=3.933	<0.0001	0.0021	Yes
Age & TC (high vs low; Table 6; Model 1)	z=0.507	0.6122	0.7385	No
Age & TC (high vs low; Table 6; Model 2)	z=0.836	0.4030	0.5469	No
Age & TC (high vs low; Table 6; Model 3)	z=1.275	0.2023	0.3810	No
Age & TC (high vs low; Table 6; Model 4)	z=1.243	0.2140	0.3810	No
Age & HDL-C (high vs low; Table 6; Model 1)	z=2.400	0.0164	0.0577	No
Age & HDL-C (high vs low; Table 6; Model 2)	z=2.297	0.0216	0.0714	No
Age & HDL-C (high vs low; Table 6; Model 3)	z=1.894	0.0583	0.1735	No
Age & HDL-C (high vs low; Table 6; Model 4)	z=1.885	0.0594	0.1735	No
Age & HbA1c (high vs low; Table 8; Model 1)	z=1.284	0.1991	0.3810	No
Age & HbA1c (high vs low; Table 8; Model 2)	z=1.290	0.1969	0.3810	No
Age & HbA1c (high vs low; Table 8; Model 3)	z=1.132	0.2574	0.4076	No
Age & HbA1c (high vs low; Table 8; Model 4)	z=1.073	0.2831	0.4350	No
Age & GLU (high vs low; Table 8; Model 1)	z=-0.297	0.7663	0.8756	No
Age & GLU (high vs low; Table 8; Model 2)	z=-0.275	0.7834	0.8756	No
Age & GLU (high vs low; Table 8; Model 3)	z=-0.046	0.9637	0.9897	No
Age & GLU (high vs low; Table 8; Model 4)	z=0.000	0.9996	0.9996	No
Age & CRP (high vs low; Table 8; Model 1)	z=2.608	0.0091	0.0461	Yes

Comparison	z (approx.)	Unadjusted p	BH adjusted p	Significant after FDR
Age & CRP (high vs low; Table 8; Model 2)	z=2.648	0.0081	0.0461	Yes
Age & CRP (high vs low; Table 8; Model 3)	z=1.573	0.1156	0.2921	No
Age & CRP (high vs low; Table 8; Model 4)	z=1.550	0.1210	0.2921	No
Age & BMI (high vs low; Table 10; Male; Model 1)	z=0.059	0.9533	0.9897	No
Age & BMI (high vs low; Table 10; Female; Model 1)	z=-0.156	0.8759	0.9246	No
Age & Pulse rate (high vs low; Table 10; Male; Model 1)	z=1.427	0.1537	0.3245	No
Age & Pulse rate (high vs low; Table 10; Female; Model 1)	z=0.898	0.3691	0.5195	No
Age & Height (high vs low; Table 10; Male; Model 1)	z=-2.989	0.0028	0.0236	Yes
Age & Height (high vs low; Table 10; Female; Model 1)	z=0.023	0.9820	0.9951	No
Age & Weight (high vs low; Table 10; Male; Model 1)	z=1.498	0.1342	0.3000	No
Age & Weight (high vs low; Table 10; Female; Model 1)	z=1.186	0.2356	0.3810	No
Age & LDL-C (high vs low; Table 10; Male; Model 1)	z=0.749	0.4538	0.5846	No
Age & LDL-C (high vs low; Table 10; Female; Model 1)	z=3.195	0.0014	0.0133	Yes
Age & TG (high vs low; Table 10; Male; Model 1)	z=1.206	0.2278	0.3810	No
Age & TG (high vs low; Table 10; Female; Model 1)	z=3.432	0.0006	0.0114	Yes
Age & TC (high vs low; Table 10; Male; Model 1)	z=0.614	0.5394	0.6720	No
Age & TC (high vs low; Table 10; Female; Model 1)	z=0.417	0.6765	0.8033	No
Age & HbA1c (high vs low; Table 10; Male; Model 1)	z=-1.066	0.2862	0.4350	No

Comparison	z (approx.)	Unadjusted p	BH adjusted p	Significant after FDR
Age & HbA1c (high vs low; Table 10; Female; Model 1)	z=2.616	0.0089	0.0461	Yes
Age & GLU (high vs low; Table 10; Male; Model 1)	z=-2.576	0.0100	0.0466	Yes
Age & GLU (high vs low; Table 10; Female; Model 1)	z=3.239	0.0012	0.0130	Yes
Age & HDL-C (high vs low; Table 10; Male; Model 1)	z=2.042	0.0412	0.1305	No
Age & HDL-C (high vs low; Table 10; Female; Model 1)	z=0.885	0.3764	0.5201	No
Age & TRL-C (high vs low; Table 10; Male; Model 1)	z=1.017	0.3090	0.4605	No
Age & TRL-C (high vs low; Table 10; Female; Model 1)	z=1.242	0.2144	0.3810	No
Age & CRP (high vs low; Table 10; Male; Model 1)	z=0.248	0.8045	0.8861	No
Age & CRP (high vs low; Table 10; Female; Model 1)	z=3.891	<0.0001	0.0019	Yes

Notes: Overall family includes all interaction tests reported in Table 4, Table 6, Table 8, and Table 10. Benjamini–Hochberg (BH) adjusted p-values were computed across the overall family based on raw interaction p-values. For raw p-values reported as "<0.0001", a value of 0.0001 was used for BH calculations (displayed as "<0.0001" in the table). Test statistics are reported as z (approx.) values derived from two-sided p-values ($z = \text{sign}(\Delta\beta)\times|\text{qnorm}(p/2)|$), where sign(Δβ) is determined by the direction of the difference in age slopes between high and low strata within the corresponding model/sex. For p-values reported as "<0.0001", the corresponding z (approx.) should be interpreted as a lower bound. Significant after FDR indicates BH adjusted p-value <0.05.

Supplementary Table S2. CRP Sensitivity Analysis: Exclude Participants with CRP>10 mg/L

Sex	CRP group	N (original)	Excluded (n)	Excluded (%)	N (after)
Female	High (>=5 mg/L)	26	3	11.54	23

Female	Low (<5 mg/L)	225	0	0.00	225
Male	High (>=5 mg/L)	26	8	30.77	18
Male	Low (<5 mg/L)	237	0	0.00	237
Overall	High (>=5 mg/L)	52	11	21.15	41
Overall	Low (<5 mg/L)	462	0	0.00	462
Overall	All	514	11	2.14	503

Note: All exclusions occurred in the High CRP group; no Low CRP participant had CRP > 10 mg/L.

Supplementary Table S3. CRP Sensitivity Analysis: CRP distribution before and after exclusion

Subgroup	Pre N	Pre mean (SD)	Pre median [IQR]	Pre range	Post N	Post mean (SD)	Post median [IQR]	Post range
Overall	514	2.57 (2.25)	1.78 [1.30, 3.00]	0.25–16.57	503	2.34 (1.63)	1.76 [1.29, 2.81]	0.25–9.81
High CRP (Overall)	52	7.95 (3.04)	6.88 [5.66, 9.30]	5.04–16.57	41	6.59 (1.32)	6.27 [5.58, 7.41]	5.04–9.81
Low CRP (Overall)	462	1.97 (1.00)	1.63 [1.24, 2.39]	0.25–4.96	462	1.97 (1.00)	1.63 [1.24, 2.39]	0.25–4.96
Male - High CRP	26	8.59 (3.05)	7.46 [5.79, 10.50]	5.23–15.24	18	6.88 (1.46)	6.78 [5.61, 7.48]	5.23–9.41
Male - Low CRP	237	2.10 (1.00)	1.78 [1.35, 2.66]	0.73–4.96	237	2.10 (1.00)	1.78 [1.35, 2.66]	0.73–4.96
Female - High CRP	26	7.31 (2.95)	6.31 [5.60, 7.58]	5.04–16.57	23	6.37 (1.19)	6.04 [5.51, 6.97]	5.04–9.81

Female - Low CRP	225	1.83 (0.97)	1.53 [1.17, 2.13]	0.25–4.88	225	1.83 (0.97)	1.53 [1.17, 2.13]	0.25–4.88

Supplementary Table S4. Cut-off values used for subgroup definitions

Indicator	Overall-sample cut-off (Low vs High)	Male subgroup range (Low vs High)	Female subgroup range (Low vs High)
Pulse rate (bpm)	≤76 vs >76	50–78 vs 79–103	52–79 vs 80–99
Height (cm)	≤166.5 vs >166.5	157–173 vs 173.5–195	144.5–160.5 vs 161–179.5
Weight (kg)	≤71.2 vs >71.2	55.1–81.9 vs 82.1–137.1	43.6–63.9 vs 64.0–136.5
BMI (kg/m²)	≤26.60 vs >26.60	15.31–28.07 vs 28.12–45.83	14.80–25.43 vs 25.47–39.07
Waist circumference (cm)	≤89.0 vs >89.0	59.9–90.1 vs 90.2–140.0	60.0–90.3 vs 90.5–134.6
Hip measurement (cm)	≤99.0 vs >99.0	-	-
LDL-C (mmol/L)	<1.79 vs ≥1.79	0.32–1.74 vs 1.79–3.78	0.04–2.05 vs 2.08–3.56
TG (mmol/L)	≤1.70 vs ≥1.71	0.39–1.15 vs 1.16–21.7	0.06–1.54 vs 1.56–8.31
Total cholesterol (mmol/L)	≤5.17 vs ≥5.18	2.21–5.18 vs 5.19–8.51	2.31–5.08 vs 5.09–8.19
HbA1c (%)	≤5.0 vs ≥5.1	4.3–5.0 vs 5.1–10.0	4.0–5.6 vs 5.7–8.1
Fasting glucose (mmol/L)	≤6.10 vs ≥6.11	3.94–6.10 vs 6.11–18.24	3.82–6.10 vs 6.12–15.43
HDL-C (mmol/L)	≤1.29 vs ≥1.30	0.52–1.29 vs 1.30–2.14	0.68–1.29 vs 1.30–2.94
TRL-C (mmol/L)	≤0.56 vs ≥0.57	0.02–0.56 vs 0.57–4.88	0.17–0.56 vs 0.57–5.00
CRP (mg/L)	<5.0 vs ≥5.0	0.25–4.96 vs 5.04–16.57	0.25–4.96 vs 5.04–16.57

Notes. (1) Table S4 summarizes the subgroup cut-off definitions applied in the interaction analyses. (2) Anthropometric indicators were categorized into low and high subgroups using sample-based cut-off values to compare age–CAVI associations across relative body-size strata within the analytic sample. (3) Biochemical indicators were categorized using indicator-specific cut-off values to preserve consistency with the original data-processing scheme and, where applicable, clinical or laboratory interpretability. (4) In sex-stratified analyses, sex-specific subgroup ranges were applied separately within men and women to account for sex-related differences in the distributions of the indicators. (5)

These subgroup definitions were used for descriptive interaction analyses and visualization only and were not intended to represent clinical diagnostic thresholds unless the corresponding indicator had an established laboratory or clinically meaningful boundary.
